# Supplementary material for: Optimal planning and allocation of Plug-in Hybrid Electric Vehicles charging stations using a novel hybrid optimization technique
Source: PLoS One. 2023 Jul 26;18(7):e0284421. doi: 10.1371/journal.pone.0284421 (PMC10370702; doi:10.1371/journal.pone.0284421)
Supplement: Attachment — Submitted filename: ResponsetoReviewerComments_PlosOne.docx [file pone.0284421.s006.docx]

**RESPONSE TO REVIEWER COMMENTS**

**Manuscript No.** - PONE-D-22-29934

**Manuscript Title** - Optimal Planning and Allocation of Plug-in Hybrid Electric Vehicles Charging Stations using a Novel Hybrid Optimization Technique

**Authors** - Ayyappan Subramaniam & Lal Raja Singh Ravi Sing

**Reviewer #1:**

This paper presents the Optimal Planning and Allocation of Plug-in Hybrid Electric Vehicles Charging Stations using a Novel Hybrid Optimization Technique. The paper can be accepted by addressing the following issues.

1. Please ensure all the variables have been defined correctly.

In the revised manuscript, variables are defined as per the comments of the reviewer.

2. The reinforcement learning has been widely used for HEV and energy system optimization, e.g., DOI: 10.1109/TIE.2021.3070514; DOI: 10.1109/TII.2020.3014599. Such relevant works can be included for completeness and to enhance the literature review.

Based on the reviewer comments, the specified two literatures have been included in the literature review to enhance the review section. It is included in reference section at Sl.No. 34 & 35.

3. Please explain more about the validation condition.

Based on the reviewer comments, the validation condition has been detailed in the revised manuscript at the performance analysis section.

4 The figures are not in good quality. Please modify.

In respect of the reviewer comments, the quality of the Figures is improved in the revised manuscript submitted.

5. The conclusion can be improved by giving the primary findings with necessary statistical results.

Primary findings are included and the conclusion section is revised as per the reviewer comments.

**Reviewer #2:**  Some concerns are listed below for possible further improvement of this paper.

1. The number of relevant literature introductions is relatively small, and half of them are articles published in 2018. It is suggested to add some new articles.

With respect to the reviewer comments, new literatures pertaining to 2021 to 2023 has been included in detail in the Introduction section. These are also included in the references from Sl.no. 36 - 49.

//The contents included based on reviewer comments are shown in RED colour in the revised manuscript//

2. The introduction of relevant literature should be classified according to some logic, rather than simply spread out according to time.

Yes, based on the reviewer comments, the literature section is remodified in section 1 of the revised manuscript.

3. How to ensure that the power grid can not only withstand the disorderly charging of charging piles but also ensure the charging efficiency?

This is because the voltage profile will be maintained substantially due to which the losses will be minimized increasing the charging efficiency. The power loss minimization enables to reduce the energy consumption and thereby the efficiency gets increased.

4. How to calculate cost, VRPindex and Aindex in the objective function.

Equation (5) in the revised manuscript, is employed to evaluate the necessary parameters.

5. Please analyze the experimental results according to the principles of AT-AQ and other comparison algorithms, and explain why AT-AQ can achieve better experimental results.

Based on the reviewer comments, the analysis is detailed in the revised manuscript at the performance analysis section.

6. Can you give the specific regional distribution of charging stations?

The dataset employed for testing and validating the proposed optimization model pertains to the usage of electric vehicles within the campus of Georgia Tech, Atlanta, USA and the vehicles were charged at the conference centre parking station and around 150 vehicles were flying around the campus. The average driving distance of the vehicles is 31 km. Thus the regional distribution will be around the campus of Georgia Tech, Atlanta, USA in this research study.

7. Noting that this study focuses on PHEV, is the proposed method applicable to EV charging in the future?

Yes, the proposed approach is suitable for both PHEV and EV. The proposed optimization model is generic and shall be applied for both PHEV and EV due to their generalization ability and learning ability.

**Reviewer #3:**

1- Captions of most figures are inappropriate. Authors should give each figure an independent caption.

Based on the reviewer comments, the captions of the figures are changed in the revised manuscript.

2- Grammar errors and typos should be avoided. Many words’ first letter is improperly capitalized.

Based on reviewer comments, English grammatical corrections are done.

3- The literature review is suggested to be merged to Section 1.

Considering the reviewer comments, the literature review section is merged with the Introduction section in the revised manuscript.

4- Most figures are of low quality.

In the revised manuscript, the quality of figures are improved.

5- As it is stated, “the maximum produced power of EM is limited to thirty kilowatts due to battery health.” Why? More detailed explanations should be given.

This is only for the reparative braking mechanism and the conditions of the battery is primarily important for this scenario. Due to which, for this braking mechanism only the maximum power produced is limited and for other forms of braking employed it can be extended to higher kilowatts.

6- In Eqn. (5), objectives are with different units and magnitude orders. I do not think it is correct to merge them simply.

Based on the reviewer comments, in the revised manuscript, equation (5) provides the complete objective function employed in the proposed technique.

7- What are the constraints of the EV, AC/DC, and power plant in your system?

The constraints in respect of the EVs shall be AC power and necessary inverter circuits shall be employed for conversion of AC to DC and the DC power shall be stored in the batteries. Making the EVs into the power grid shall result in voltage drop, energy loss and affecting the peak load of the system. Thus, the constraints in respect of the EVs include,

- Cost of installation of the charging stations
- Increased distribution system power loss
- Difficulty in connecting the EVs for charging directly to the grid
- Problem in the source of electrical energy at a unity power factor and the voltage profile not maintained due to the power system module
- Increased power losses
- Active power loss of the distributed power system network

The above are the specific constraints pertaining to the effective location of charging stations for the Electric Vehicles.

//The above contents are included in the section 3.1 of the revised manuscript based on the reviewer comments//

8- Comparative results in Section 5 should be fully discussed and analyzed.

Based on the reviewer comments, validation is included in the performance analysis section.

9- EVs may leave the station after charging. However, EV arrival distribution during the experimental period is not introduced and considered.

EV arrival distribution is always on the first come first serve basis and based on the free charging connectors. And this initialized in the algorithm as the node that arrives will be set to 1 and the charging station shall be allocated for the same.

10- In P15, “The analysis was carried out in two different scenarios.” However, in P17, three scenarios are utilized. Moreover, what are the specific differences among these scenarios?

Three scenarios have been employed for this study, the scenarios include,

Scenario 1: Optimal placement based on distribution system conjunction with transportation system

Scenario 2: Optimal placement based on distribution generators with previous optimal charging load

Scenario 3: Allocation of distribution generators and charging station in distribution system optimally based on earlier optimal load

The authors than the reviewers and the editor for their valuable comments in improving the quality of the manuscript. Thank you.

Sincerely,

Authors
